# Supplementary material for: Large-scale cryptic proteome mining revealed potential phage-mediated host-pathogen genetic exchange in Mycobacterium tuberculosis
Source: PLoS One. 2026 May 18;21(5):e0348602. doi: 10.1371/journal.pone.0348602 (PMC13183242; doi:10.1371/journal.pone.0348602)
Supplement: S1 Fig — (DOCX) [file pone.0348602.s001.docx]

**Title: Large-scale cryptic proteome mining revealed potential phage-mediated host-pathogen genetic exchange in *Mycobacterium tuberculosis***

**Supplementary S1 Figure**

**S1 Fig:** UMAP 3D projections in the form of coordinates were subjected to K-means clustering with increasing K values, and inertia (a measure of cluster compactness) was recorded for each K value.
